# Inflammatory fibroid polyp of the small intestine presenting as small bowel obstruction with intussusception: a case report

**DOI:** 10.1093/jscr/rjad695

**Published:** 2024-01-04

**Authors:** Cian Hehir, Gavin Calpin, Gavin Dowling, Chloe Spillane, Clive Kilgallen, Arnold D K Hill

**Affiliations:** Department of Surgery, Beaumont Hospital, Beaumont Road, Dublin 9, D09 V2N0, Ireland; Department of Surgery, Royal College of Surgeons in Ireland, 123 St Stephen's Green, Dublin 2, D02 YN77, Ireland; Department of Surgery, Beaumont Hospital, Beaumont Road, Dublin 9, D09 V2N0, Ireland; Department of Surgery, Royal College of Surgeons in Ireland, 123 St Stephen's Green, Dublin 2, D02 YN77, Ireland; Department of Surgery, Beaumont Hospital, Beaumont Road, Dublin 9, D09 V2N0, Ireland; Department of Surgery, Royal College of Surgeons in Ireland, 123 St Stephen's Green, Dublin 2, D02 YN77, Ireland; Department of Surgery, Beaumont Hospital, Beaumont Road, Dublin 9, D09 V2N0, Ireland; Department of Surgery, Royal College of Surgeons in Ireland, 123 St Stephen's Green, Dublin 2, D02 YN77, Ireland; Department of Histopathology, Beaumont Hospital, Beaumont Road, Dublin 9, D09 V2N0, Ireland; School of Medicine, Royal College of Surgeons in Ireland, 123 St Stephen's Green, Dublin 2, D02 YN77; Department of Surgery, Beaumont Hospital, Beaumont Road, Dublin 9, D09 V2N0, Ireland; Department of Surgery, Royal College of Surgeons in Ireland, 123 St Stephen's Green, Dublin 2, D02 YN77, Ireland; School of Medicine, Royal College of Surgeons in Ireland, 123 St Stephen's Green, Dublin 2, D02 YN77

**Keywords:** adult intussusception, bowel obstruction, inflammatory fibroid polyp, spindle-cell

## Abstract

Inflammatory fibroid polyps (IFP) are rare benign neoplasms most commonly occurring within the respiratory tract but are rarely also observed in the gastro-intestinal tract. Herein we present the case of a 73-year-old female presenting with ileo-ileal intussusception secondary to IFP. The patient was treated with emergency laparotomy with segmental bowel resection and primary anastomosis. Histopathological analysis of the excised bowel segment initially revealed a low-grade, mural based spindle cell neoplasm with surrounding benign, reactive lymphadenopathy. Immunohistochemical analysis demonstrated that the lesional cells stained positive for Vimentin, Smooth Muscle Actin (SMA), and CD34. On secondary analysis of the specimen, the morphology and immunohistochemical profile of the mass was in keeping with IFP. No invasive malignancy was identified. Such cases have been previously reported under the pseudonym *‘the great mimicker’*, due to their striking similarity to malignant processes. This case report aims to add to the small body of research reporting such atypical presentations.

## Introduction

Intussusception, meaning the invagination of a proximal portion of intestine into a distal portion, is a common cause of bowel obstruction among the paediatric population but represents only 1% of bowel obstruction within adults [1]. Furthermore, the typically benign aetiology of intussusception in paediatrics contrasts that of adults, where over 60% of cases are tumour related—a further 50% of which represent a malignant neoplasm [2]. Of note, the risk of malignancy associated with enteric intussusception (17%), has been found to be significantly less than that of colonic (48%) [3]. Hence, the treatment of choice in adults is typically resection without reduction.

Inflammatory fibroid polyp (IFP) is a rare benign neoplasm of mesenchymal origin of uncertain aetiology. These tumours are characterized by proliferation of fibrous tissue accompanied by infiltration by various inflammatory cells, most commonly spindle cells and eosinophils [4]. IFP displays a female preponderance (1.3–1) and most commonly affects individuals in their fifth decade of life [5]. Localized infection, allergic reaction, and autoimmune processes have been previously postulated as causative mechanisms but without definitive findings [6]. When occurring within the gastrointestinal system IFP most commonly affects the stomach, small intestine, followed by large intestine in descending order [5]. As such, the presentation can be varied, ranging from gastric outlet obstruction to intussusception. However, the symptomatology of presentation is largely similar regardless of location with patients most commonly complaining of abdominal pain, nausea, and vomiting in both the acute and subacute setting [5]. With regards to immunohistochemistry, the profile of IFP is varied but most commonly stains positive for CD34, vimentin, and CD117.[7]. Herein we present the case of a 73-year-old female presenting with ileo-ileal intussusception secondary to IFP.

## Case report

**Figure 1:**
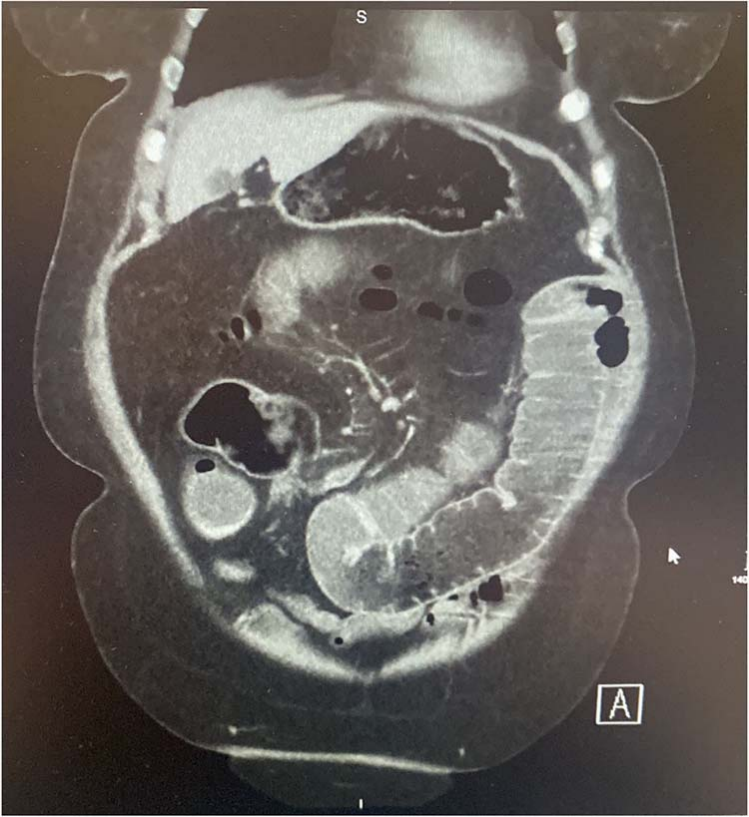
Coronal section of contrast CT AP demonstrating small bowel intussusception with proximal dilation and associated filling defect.

**Figure 2:**
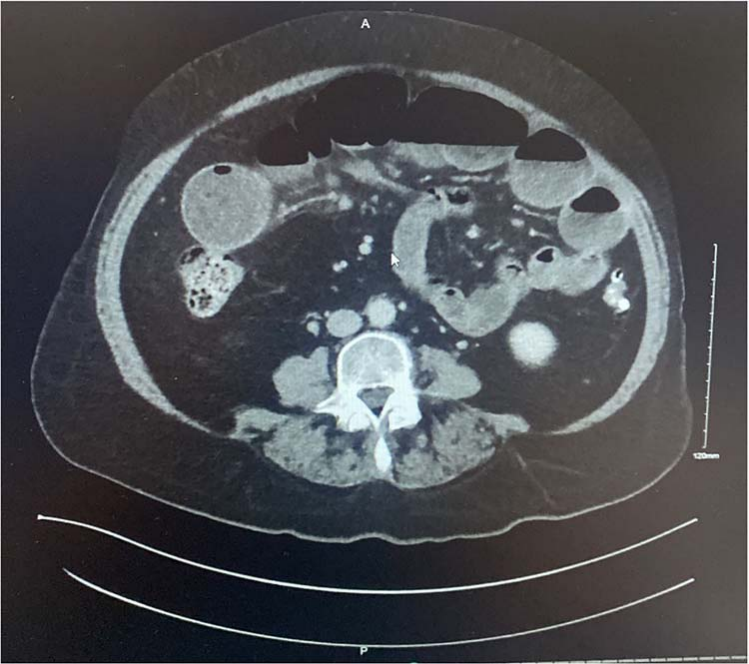
Axial section of contrast enhanced CT AP demonstrating intussusception with surrounding lymphadenopathy.

A 73-year-old female presented complaining of acute onset right sided upper abdominal pain associated with three episodes of bilious vomitus and constipation. Clinically, her abdomen was tender, distended, and tympanic with increased bowel sounds. Of note, she described a 3-month history of repeated low grade abdominal pain with vomiting. The clinical picture was concerning for that of bowel obstruction. Contrast enhanced CT scan of the abdomen and pelvis (CTAP) demonstrated multiple dilated loops of small bowel measuring 4.5 cm at their maximum calibre. In the right abdomen there was a focus of ileo-ileal intussusception with a filling defect measuring ~6–7 cm (Figs 1–2). Multiple enlarged enhancing lymph nodes, measuring up to 10 mm, were identified at the level of the intussusception. The duodenum, distal ileum, and jejunum were collapsed.

**Figure 3:**
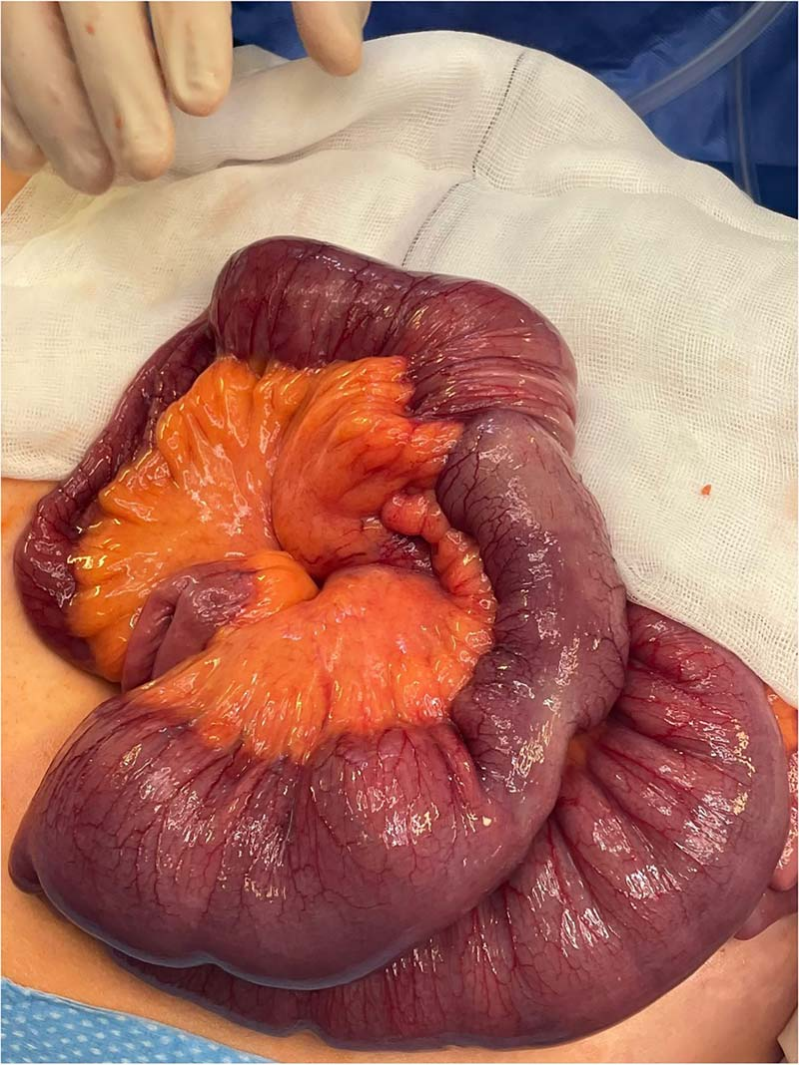
Intraoperative identification of intussusception located 1 m from the ileocecal valve.

**Figure 4:**
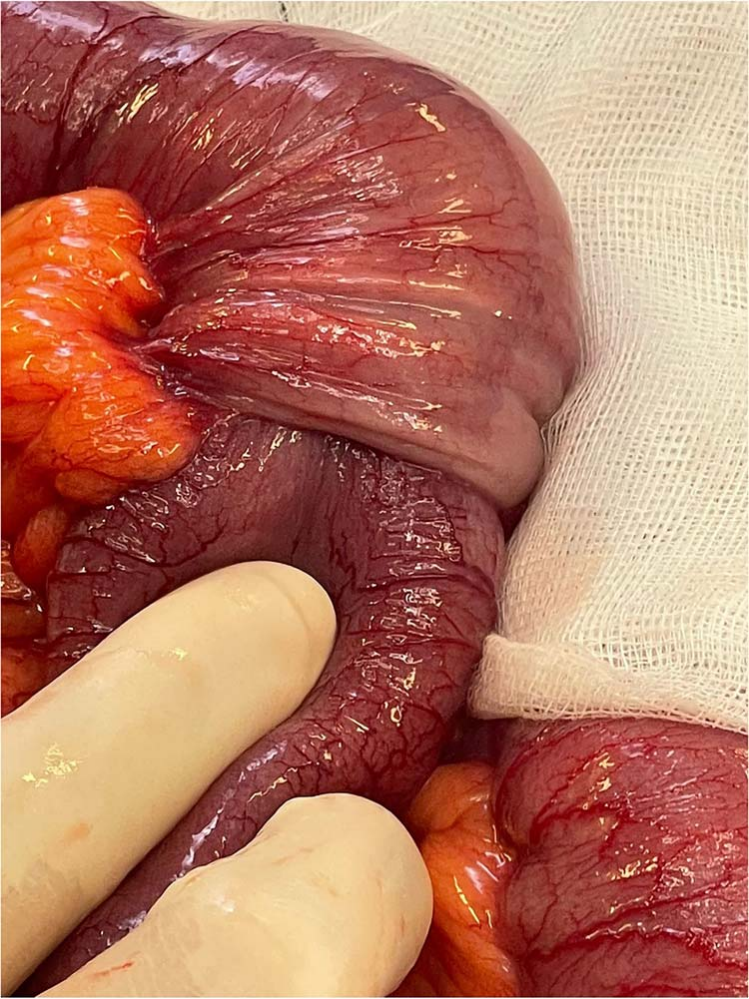
Focus of intussusception with proximal dilation and hypertrophy.

The bowel was decompressed with wide bore nasogastric tube insertion. The patient received resuscitation with IV fluids and was transferred to theatre and underwent emergency laparotomy where intussusception was identified 1 m from the ileocecal valve (Fig. 3) with proximal dilation and hypertrophy (Fig. 4). Thereafter, segmental ileal resection was carried out with stapled side-to-side anastomosis. The anastomosis was reinforced using a monofilament cross stitch. The patient returned to the ward where early enteral feeding was commenced. The postoperative period was uncomplicated with the patient opening her bowels on the third postoperative day and later discharged on the sixth postoperative day.

**Figure 5:**
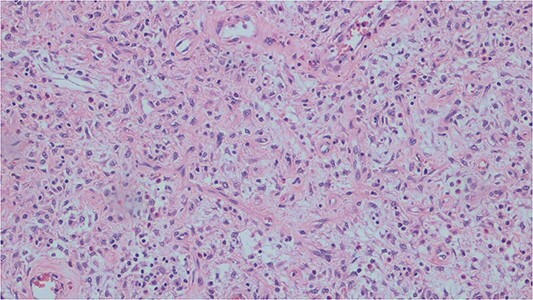
Histopathological slide demonstrating bland mesenchymal cells with a rich eosinophilic infiltrate.

**Figure 6:**
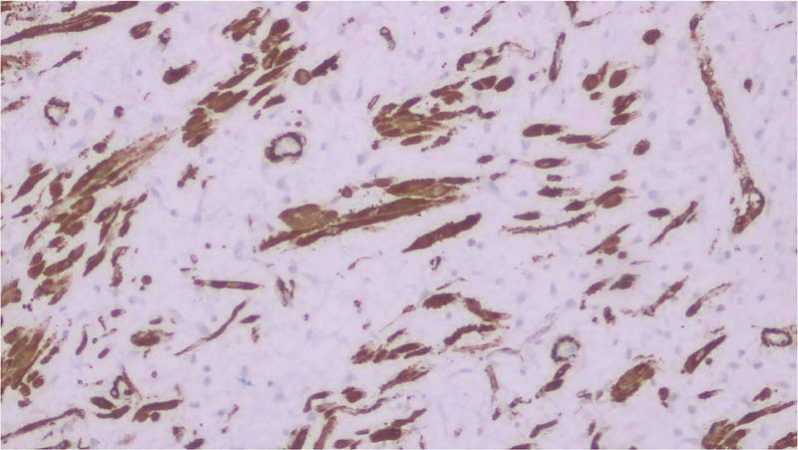
IFP cells staining positive for Vimentin.

Histopathological analysis of the excised bowel segment revealed a low-grade, mural based spindle cell neoplasm (Fig. 5) with surrounding benign, reactive lymphadenopathy. The sample was sent for further immunohistochemical analysis for definite subtyping which demonstrated that the lesional cells stained positive for Vimentin, SMA and CD34. Desmin, S100, cytokeratin DOG-1, C-KIT, and ALK-1 were negative (Fig. 6). These findings were consistent with IFP in terms of morphology and immunohistochemical profile.

## Discussion

Intussusception is a rare in adults, accounting for only 1% of bowel obstructions [1]. Of this small cohort however, a pathological lesion is found to act as a lead point in >90% of cases [2]. Such pathological lesions range from benign strictures to malignant tumours with the latter comprising of a concerningly high proportion of intussusception in adults—40% and 17% in the large and small bowel respectively. Differentiating between benign and malignant causes of intussusception can be challenging both pre-operatively and intra-operatively as well as from a histopathological perspective as seen in this case. IFP has been previously termed *‘the great mimicker’* due to its close resemblance to invasive masses such as spindle cell tumours [8] which carry a 40%–50% mortality rate when found in the small bowel [9]. Immunohistochemistry provides a useful means of definite subtyping with most IFP lesions staining positive for CD34 and, to a lesser extend vimentin and CD117 [7, 10, 11]. However, no immunohistochemical stain has proven effective in definitively identifying IFP to date.

Diagnosis of intussusception using computerized topography has shown sensitivity close to 100% [12]. However, there are no distinctive radiological features which can delineate benign causes such as IFP from the malignant. Hence, due to this diagnostic uncertainty coupled with significant risk of malignancy surgical en-bloc resection remains the mainstay in treatment when IFP occurs within the small and large bowel. The exception to this is when IFP occurs within the gastric mucosa, in which endoscopic resection is favoured [5].

IFP can present in a diagnostically challenging manner. There is a lack of reporting of such benign tumours and their varied presentations within the small bowel from a surgical, histopathological and immunohistochemical perspective. This case report aims to add to the small body of research reporting on this topic.
